# Editorial: Importance of cytokines and receptor members from the IL-1 family in the context of chronic autoimmune inflammatory diseases

**DOI:** 10.3389/fimmu.2022.974261

**Published:** 2022-07-19

**Authors:** Roba M. Talaat, Ashraf A. Tabll, Amira M. Gamal-Eldeen, Remo C. Russo

**Affiliations:** ^1^ Molecular Biology Department, Genetic Engineering and Biotechnology Research Institute (GEBRI), University of Sadat City (USC), Sadat City, Egypt; ^2^ Microbial Biotechnology Department, Biotechnology Research Institute, National Research Centre, Cairo, Egypt; ^3^ Egypt Center for Research and Regenerative Medicine, Cairo, Egypt; ^4^ Clinical Laboratory Sciences Department, College of Applied Medical Sciences, Taif University, Taif, Saudi Arabia; ^5^ High Altitude Research Center, Prince Sultan Medical Complex, Al-Hawiyah, Taif University, Taif, Saudi Arabia; ^6^ Laboratory of Pulmonary Immunology and Mechanics, Department of Physiology and Biophysics, Institute of Biological Sciences, Universidade Federal de Minas Gerais (UFMG), Belo Horizonte, Minas Gerais, Brazil

**Keywords:** IL-1, chronic inflammation, autoimmunity, COVID-19, periodontitis

The history of the IL-1 cytokine family begins in 1974 with the discovery of the IL-1α and IL-1β, described as two distinct ‘leukocytic pyrogens’, since these proteins promote fever. Today, almost 50 years after its discovery, the IL-1 cytokine family comprises 11 cytokine members encoded by 11 distinct genes in humans and mice (1–4). This family displays a complex regulation that amplify the mechanisms orchestrated by IL-1 members during acute and chronic inflammation (5–7). Recently, evidences have proposed that members of the IL-1 members are closely associated with the abnormal inflammatory and immune responses during chronic autoimmune diseases. Several immunity sensors, evolved to assist host defense by stimulating the IL-1 pathway (2, 5, 6). Regarding the understanding of the IL-1 cytokine family over pasting years, it mainly showed pro-inflammatory activities (IL-1α, IL-1β, IL-18, IL-33, IL-36α, IL-36β, and IL-36γ) but also contained four members that suppress inflammation: two specific receptor antagonists (IL-1Ra and IL-36Ra), and less studied, two members that broadly suppress cytokines and chemokines during innate inflammation (IL-37 and IL-38) (1–8).

This Research Topic cover the recent advances in the role of several IL-1 family members, with particular emphasis on newly discovered IL-36, IL-37, and IL-38, in several autoimmune inflammatory diseases of the skin, gut, and teeth. The authors also discussed the role of cytokine gene polymorphism in disease susceptibility and pathogenesis. With the spread of COVID-19 infection worldwide and the ultimate need for vaccination, the efficacy and safety of COVID-19 vaccination in autoinflammatory rheumatic diseases under immunosuppressive drugs was discussed.

Periodontitis is a chronic inflammatory illness that causes tooth loss by destroying the tooth-supporting tissues (9), and cytokines are involved in periodontitis (10). Liu and Li looked at 147 publications with polymorphisms in 12 interleukins [Th1 (IL-2, IFN-γ, and TNF-α), Th2 (IL-4 and IL-13), Th17 (IL-1α, IL-1β, IL-6, and IL-17), and Treg cytokines (IL-10 and TGF-β). Polymorphisms in the Th2 cytokine family, such as IL-4 and IL-13, have not been linked to periodontitis pathogenesis. However, Th17 cytokine genetic polymorphism may be a risk factor for periodontitis, while Treg cells may have a protective role in preventing periodontal inflammation. In this sense, IL-1 is an important cytokine biomarker in the development and progression of periodontitis.

Chronic inflammatory diseases may emerge in various tissues, including the skin, due to an imbalance in pro-and anti-inflammatory cytokines from the IL-1 family (11–14). Martin et al. summarize the recent advances in the biology of anti-inflammatory members of the IL-1 cytokine family and their roles in controlling inflammatory responses in human and mouse skin. Martin et al. reported that IL-1Ra, IL-36Ra, IL-37, and IL-38, are constitutively expressed in keratinocytes and exert their regulatory roles in skin inflammation, suggesting new ways to treat inflammatory skin diseases.

IL-37, commonly known as IL-1 family member 7 (IL-1F7) (15, 16) show anti-inflammatory properties (17–19), and is considered as a disease biomarker in various inflammatory and autoimmune disorders (20). Santarelli et al. established the first reference range of circulating IL-37 plasma and serum levels in healthy adult humans. Santarelli et al. established a tentative reference range for circulating IL-37 in healthy people. Large, multi-ethnic, healthy population studies are needed to obtain a credible clinical reference range. They also recommended further investigations into demographics, sample matrices, collection, processing, and storage procedures that affect IL-37 detection levels.

With the spread of COVID-19 worldwide, the vaccination is crucial to ending the pandemic and preventing mortality. The immunosuppression established during treatment autoimmune rheumatic diseases (AIIRD) by IL-1 blockage may compromise the immunization process (21, 22). Atagündüz et al. summarize all forms of vaccinations under IL-1 blockade discussing the use and timing of COVID-19 vaccination in patients receiving anti-IL-1 therapy. Atagündüz et al. reported that COVID-19 vaccine efficacy might be lower in patients treated with IL-1 blockade. As noted by Atagündüz et al., COVID-19 develops more rapidly in untreated patients. Atagündüz et al. recommended that patients under IL-1 blockade be vaccinated without interrupting anti-cytokine therapy, especially those patients with ongoing high disease activity, to avoid disease relapses.

As a member of the IL-1 cytokine family, IL-36 cytokines include the anti-inflammatory cytokine IL-36Ra and the pro-inflammatory cytokines IL-36α, IL-36β, and IL-36γ (23). The most of the research has focused on its involvement in autoimmune diseases (24), but have demonstrated in the lung inflammation during infectious and non-infectious disorders (25–27). Peñaloza et al. review the current literature concerning the biology of IL-36 cytokines, including their synthesis and activation, also their impact on myeloid and lymphoid cells during inflammation. They also discussed their function during bacterial and viral lung infections, and other inflammatory lung disorders such as cancer, cystic fibrosis, allergic asthma, chronic obstructive pulmonary disease, and lung fibrosis. Peñaloza et al. also outline recent therapeutic development targeting the IL-36 pathway and the potential to treat lung inflammatory disorders.

The gastrointestinal tract (GIT) is lined by a single cell layer of intestinal epithelial cells (IECs) which are interconnected by the tight junctions (TJs), which serve as a gate or barrier to paracellular permeation of luminal contents (28). Disruption of the intestinal TJ barrier plays an essential role in the pathogenesis of several inflammatory conditions of the gut, including celiac disease and inflammatory bowel disease (IBD), characterized by elevated levels of IL-1 (28–30). In this review, Kaminsky et al. review most of the published studies that deal with the clinical consequences of IL-1β intestinal barrier regulation in intestinal inflammation, the underlying mechanisms and intracellular signaling pathways involved in TJ barrier permeability modulation, and the downstream molecular targets of IL-1β. Kaminsky et al. also discussed the potential therapeutic targeting of the TJ barrier.

In conclusion, this Research Topic highlights recent findings and new insights into the most recent development of the IL-1 family in the pathophysiology of several autoimmune diseases. The current research may provide new insight and advancements for future research targeting IL-1 in autoimmune diseases affecting teeth, skin, and GIT. Finally, in this Research Topic, the authors also go through elements of clinical translation and the biology of cytokines belonging to the IL-1 family in animal models, identifying potential targets for developing novel therapeutic approaches for autoimmunity.

## Author Contributions

RT, AT, AG-E and RR performed literature research, discussed the articles, and wrote the manuscript. All authors contributed to the article revision and approved the submitted version.

## Funding

This work did not receive financial support for its design and publication. RR is fellow from National Council for Scientific and Technological Development (CNPq) [Grant number: 312839/2020-0], and the laboratory is supported by grant from Fundação de Amparo à Pesquisa do Estado de Minas Gerais (FAPEMIG), Rede Mineira de Pesquisa Translacional em Imunobiológicos e Biofármacos no Câncer (REMITRIBIC) [Grant number: RED-00031-21] and Demanda Universal 01/2021 [Grant number: APQ-02571-21].

## Conflict of Interest

The authors declare that the research was conducted in the absence of any commercial or financial relationships that could be construed as a potential conflict of interest.

## Publisher’s Note

All claims expressed in this article are solely those of the authors and do not necessarily represent those of their affiliated organizations, or those of the publisher, the editors and the reviewers. Any product that may be evaluated in this article, or claim that may be made by its manufacturer, is not guaranteed or endorsed by the publisher.
